# Blockade of Store-Operated Calcium Entry Reduces IL-17/TNF Cytokine-Induced Inflammatory Response in Human Myoblasts

**DOI:** 10.3389/fimmu.2018.03170

**Published:** 2019-01-14

**Authors:** Audrey Beringer, Yves Gouriou, Fabien Lavocat, Michel Ovize, Pierre Miossec

**Affiliations:** ^1^Immunogenomics and Inflammation Research Unit EA4130, Department of Clinical Immunology and Rheumatology, Edouard Herriot Hospital, University of Lyon, Hospices Civils de Lyon, Lyon, France; ^2^CarMeN Laboratory, University of Lyon, INSERM 1060, INRA 1397, Université Claude Bernard Lyon1, INSA Lyon, Groupement Hospitalier Est, Bron, France; ^3^Service d'Explorations Fonctionnelles Cardiovasculaires and CIC de Lyon, Hôpital Louis Pradel, Hospices Civils de Lyon, Lyon, France

**Keywords:** inflammatory myopathies, interleukin-17, tumor necrosis factor-α, store-operated calcium entry, myoblasts

## Abstract

Muscle inflammation as in idiopathic inflammatory myopathies (IIM) leads to muscle weakness, mononuclear cell infiltration, and myofiber dysfunction affecting calcium channels. The effects of interleukin-17A (IL-17) and tumor necrosis factor-α (TNFα) on inflammation and calcium changes were investigated in human myoblasts. Human myoblasts were exposed to IL-17 and/or TNFα with/without store-operated Ca^2+^ entry (SOCE) inhibitors (2-ABP or BTP2). For co-cultures, peripheral blood mononuclear cells (PBMC) from healthy donors activated or not with phytohemagglutinin (PHA) were added to myoblasts at a 5:1 ratio. IL-17 and TNFα induced in synergy CCL20 and IL-6 production by myoblasts (>14-fold). PBMC-myoblast co-cultures enhanced CCL20 and IL-6 production in the presence or not of PHA compared to PBMC or myoblast monocultures. Anti-IL-17 and/or anti-TNFα decreased the production of IL-6 in co-cultures (*p* < 0.05). Transwell system that prevents direct cell-cell contact reduced CCL20 (*p* < 0.01) but not IL-6 secretion. IL-17 and/or TNFα increased the level of the ER stress marker Grp78, mitochondrial ROS and promoted SOCE activation by 2-fold (*p* < 0.01) in isolated myoblasts. SOCE inhibitors reduced the IL-6 production induced by IL-17/TNFα. Therefore, muscle inflammation induced by IL-17 and/or TNFα may increase muscle cell dysfunction, which, in turn, increased inflammation. Such close interplay between immune and non-immune mechanisms may drive and increase muscle inflammation and weakness.

## Introduction

Idiopathic Inflammatory Myopathies (IIM) such as dermatomyositis and polymyositis are chronic muscle diseases characterized by muscle inflammation, skeletal muscle weakness and early sarcopenia. Calcium (Ca^2+^) dysregulation contributes to muscle cell dysfunction with effects on contractibility (1). Store-operate calcium entry (SOCE) is a major pathway for Ca^2+^. SOCE is activated by Ca^2+^ depletion from the endoplasmic reticulum (ER) that triggers the store-operated calcium channels (SOCs) opening through stromal-interacting molecule (STIM1) translocation to Orai channels. SOCE process is important for maintaining proper Ca^2+^ store filling that is crucial for muscle function (2). Overexpression of STIM1 and Orai1 and the associated rapid activation and deactivation of SOCE was observed in dystrophic mouse muscles (3). Moreover, SOCE activation is essential in the induction of myoblast differentiation (4). A tight regulation of SOCE is therefore required for proper muscle development and contractile function (2).

IIM are characterized by inflammatory/immune cell infiltration (5, 6), which contributes to muscle inflammation and dysfunction. Among the local secreted cytokines, Tumor necrosis factor-α (TNFα) was found upregulated in IIM samples (7–9). Interleukin (IL)-17A, also known as IL-17, was detected in lymphocytic infiltrates in myositis tissues (10, 11) and IL-17 serum level was elevated in IIM patients (12–14). In cultured human myoblasts, both IL-17 and TNFα induced massive myoblast inflammatory response (10, 15, 16). The elevated levels of IL-17 and TNFα in IIM and their *in vitro* effects suggest that these cytokines play an important role in the pathogenesis of myositis.

Here, the interplay between inflammation and Ca^2+^ dysregulation was studied in human myoblasts exposed to IL-17 and TNFα. Immune cell and myoblast co-cultures were used to mimic the immune cell infiltrate found in IIM and to assess the contribution of these cell-cell contacts. The results indicate that IL-17 and TNFα played an important role in myoblast inflammatory response especially in IL-6 secretion. ER stress, reactive oxygen species (ROS) generation and SOCE were induced by IL-17 and TNFα whereas SOCE inhibition reduced IL-6 production inducted by IL-17/TNFα. Such close interplay between immune and non-immune mechanisms may drive and increase muscle weakness.

## Materials and Methods

### Isolation and Culture of Muscle Cells

Muscle samples were obtained from subjects undergoing orthopedic surgery. Biopsies were performed on m. vastus lateralis (femoral quadriceps) at distance of the joint. Written informed consent was obtained before surgery according to the policies of the local ethical committee and the Ministry of Research, which approved the study (reference number: AC-2016-2729). After surgery, muscle samples were immediately placed in sterile PBS with antibiotics (penicillin and streptomycin, Eurobio, Courtaboeuf, France) and washed. The fat and fibrous tissues were removed. Muscle samples were cut into fragments (1–2mm^3^) and incubated at 37°C for 30 min with 1 mg/mL collagenase (Sigma-Aldrich, St Louis, MO, USA). After washing and filtration, a first selection was done to remove fibroblasts by incubating the supernatants in petri dishes at 37°C for 1 h. Unattached myoblasts were then transferred and cultured at 37°C/5% CO_2_ in Ham's-F10 medium (Eurobio) supplemented with 20% fetal bovine serum (Life Technologies, Carlsbad, USA), 2% Penicillin-Streptomycin (Eurobio), 1% L-glutamine (Eurobio), and 1% Amphotericin B (Eurobio). After 10 days, adherent cells were detached with trypsin (Eurobio), and myoblasts were purified by positive selection with CD56 microbeads (Miltenyi Biotec, Bergisch Gladbach, Germany), according to the instructions of the manufacturer. Myoblasts were used between passages 2 and 8.

### Myoblast Culture Exposures

Myoblasts were seeded at a density of 50,000 cells/cm^2^. After adhesion, cells were stimulated with 50 ng/mL IL-17A (Dendritics, Lyon, France) or 1 ng/mL TNFα (R&D Systems, Minneapolis, USA) alone or in combination. To inhibit SOCE, BTP2 (or YM58483) and 2-aminoethyl diphenylborinate (2-APB) inhibitors (Sigma-Aldrich) were used between 10 and 50 μM.

### PBMC Isolation and Co-culture Assays

Whole blood samples were obtained from the Etablissement Français du Sang. Peripheral blood mononuclear cells (PBMCs) were isolated by Ficoll-Hypaque (Eurobio) density gradient centrifugation. Cells were maintained in RPMI 1,640 medium supplemented with 10% human AB serum (Etablissement Français du Sang, La Plaine Saint-Denis, France), 2% Penicillin-Streptomycin (Eurobio) and 1% L-glutamine (Eurobio). PBMCs were activated or not with 5 μg/mL phytohemagglutinin (PHA) (Sigma-Aldrich) and added on adherent myoblasts at a ratio of 5 PBMCs for 1 myoblast. For cell culture insert assays, myoblasts were cultured at the bottom of a culture plate well and PBMCs were placed in Falcon® cell-culture inserts (Corning, NY, USA) with a small-pored membrane (0.4 μm) preventing cell-cell contacts but not the crossing of soluble factors. For the IL-17 and TNFα neutralization assays, PBMCs activated or not with PHA for 24 h were exposed to an anti-IL-17 antibody (R&D Systems) and/or the anti-TNFα antibody infliximab (Merck, Kenilworth, USA) at 10 μg/mL for 3 h before being added to the HepaRG cells.

### Enzyme-Linked Immunosorbent Assay (ELISA)

After 48 h of treatment, supernatants were harvested and the IL-6 and chemokine (C-C motif) ligand 20 (CCL20) productions were quantified with commercially available ELISA kits (R&D Systems) according to the manufacturer's instructions.

### Quantitative Real Time-PCR

Total RNA was purified using an RNeasy® Plus Mini kit (Qiagen, Hilden, Germany). cDNA was synthetized using the iScript™ kit (Bio-Rad, Hercules, CA, USA). PCR amplification was performed using the CFX96^TM^ Real time system instrument (Bio-Rad) with the iTaq^TM^ universal SYBR® green supermix (Bio-Rad) and the Qiagen QuantiTect® primers (QT00083538 for STIM1 and QT01870043 for ORAI1). The expression of the genes of interest was normalized to the expression of the housekeeping GAPDH gene.

### Cell Lysis and Western Blotting

Cell lysates were obtained by lysing cells with RIPA buffer supplemented with 1 mM Na_3_VO_4_, 1 mM DTT, 20 mM NAF, 5 mM EDTA, and a cocktail of proteases inhibitors. Total protein concentration was determined using Bicinchoninic acid method (BCA, Interchim) and 25 μg of protein of each sample was loaded on 12% sodium dodecyl sulfate polyacrylamide gel (SDS-PAGE). Migration was performed during 15 min at 90 V followed by 60 min at 130 V. Proteins were then blotted on a polyvinylidene difluoride (PVDF) membrane by electro transfer (Trans-Blot Turbo Transfer, Bio-Rad). PVDF membrane was incubated at room temperature for 1 h with 5% milk in PBS for blocking and then incubates overnight at 4°C in the same buffer with the primary antibody (Grp78, sc-376768; STIM1, ab108994; ORAI1, sc68895; Tubulin, sc-5286). Secondary Horse radish peroxidase (HRP) coupled antibodies and ECL (entry-level peroxidase substrate for enhanced chemiluminescence) plus kit and Western Blotting detection system from GE Healthcare were used to reveal the proteins. The protein amount was determined using ImageLab software (Bio-Rad).

### Wide-Field Microscopy for Ca^2+^ Live Cell Imaging

Cells were imaged on an epifluorescence microscope Leica DMI6000B using 40x objective equipped with Orca-Flash 4.0 digital camera (Hamamatsu). Myoblasts were double excited at 340 and 380 nm and emission was collected at 510 nm with identical acquisition parameters. Medium was replaced by a Calcium Containing Buffer (CCB) (in mmol/L: 140 NaCl, 5 KCl, 10 HEPES, 1 MgCl_2_, 2 CaCl_2_, 10 glucose, adjusted to pH7.4) containing 3 μmol/L of fura2-AM during 30 min at room temperature. Cells were washed twice with calcium free buffer in which 0.1 mmol/L EGTA was added and placed under the microscope. For depletion of Ca^2+^ stores, cyclopiazonic acid (CPA*)* (10 μM) was used and then 2 mM calcium solution (CCB) was added to trigger the SOCE. Fluorescence ratios were calculated in metaFluor 6.3 (Universal Imaging) and analyzed in Origin Pro (OriginLab) + GraphPad Prism 4 (GraphPad).

### Confocal Microscopy for Oxidative Stress Detection

Cells were imaged on a confocal microscope Nikon A1r using 40x objective. Myoblasts were excited at 640 nm and emission was collected at 665 nm. Medium was replaced by a CCB (in mmol/L: 140 NaCl, 5 KCl, 10 HEPES, 1 MgCl2, 2 CaCl2, 10 glucose, adjusted to pH7.4) containing 2.5 μmol/L of CellROX™ Deep Red Reagent during 30 min at 37°C. Fluorescence intensity was analyzed in ImageJ Fiji (https://fiji.sc/#, NIH). A threshold at the third quartile of the pixel intensity distribution was applied before the analysis.

### Confocal Microscopy for STIM1 Puncta Analysis

Cells were imaged on a confocal microscope Nikon A1r using 40x objective. Myoblasts were excited at 488 nm and emission was collected at 510 nm. Medium was replaced by a calcium free buffer (CFB) (in mmol/L: 140 NaCl, 5 KCl, 10 HEPES, 1 MgCl2, 0.1 EGTA, 10 glucose, adjusted to pH7.4). Cells were washed twice with calcium free buffer in which 0.1 mmol/L EGTA was added and placed under the microscope. For depletion of Ca^2+^ stores, cyclopiazonic acid (CPA*)* (10 μM) was used in order to form STIM1 puncta.

For each coverslip, 10 cells were imaged for each experiment and then analyzed with either Image J to calculate the co-localization coefficients or MATLAB® (MathWorks®) to perform Image Correlation spectroscopy (ICS).

For ICS analysis, images of fluorescence channel were filtered and transformed in binary images. The filtering threshold was calculated automatically by the algorithm and determined as the mean value of the fluorescence intensity in each image. The series of images were analyzed with a batch-ICS algorithm adapted from the FICS algorithm developed by Dr Heliot's team (17). Mean and SEM of both surface area and density of the fluorescent clusters were figured out automatically by the batch-ICS algorithm.

### Statistical Analysis

Data are presented as the mean ± SEM. Statistical differences were analyzed using the non-parametric Wilcoxon paired-test. *P*-values lower than 0.05 were considered significant.

## Results

### Synergistic Effect of IL-17 and TNFα on CCL20 and IL-6 Production by Myoblasts

**Figure 1:**
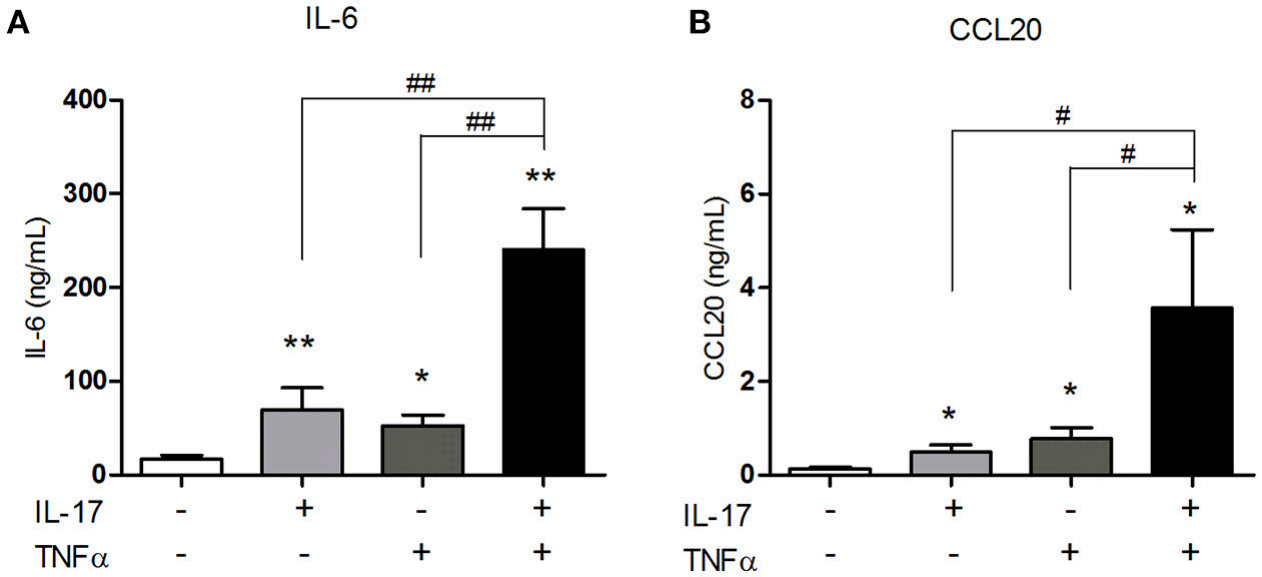
Synergistic effect of IL-17 and TNFα on the CCL20 and IL-6 production by myoblasts. Myoblasts were treated with IL-17 (50 ng/mL) and/or TNFα (1 ng/mL) for 48 h. IL-6 and CCL20 secretion by myoblasts was quantified by ELISA **(A,B)**. Data are the mean of 5–8 independent experiments ± SEM; ^*^p < 0.05, ^**^p < 0.01 vs. control untreated condition and #*p* < 0.05, ^**^p < 0.01 vs. other inflammatory conditions.

IL-6 is a pro-inflammatory cytokine involved in the differentiation of Th17 cells, the main IL-17-producing cells. By attracting Th17 cells and dendritic cells, CCL20 plays an important role in the local immune cell recruitment (18). IL-17 and TNFα are involved in IL-6 and/or CCL20 production by muscle cells (10, 15, 16) but the effect of their combination has not yet been investigated. The IL-17/TNFα effect on IL-6 and CCL20 release was studied in human myoblasts. IL-17, TNFα, and the IL-17/TNFα combination increased significantly IL-6 production by 4-, 3-, and 14-fold respectively compared to untreated condition (Figure 1A). CCL20 secretion by myoblasts was also induced by IL-17 alone (4-fold), TNFα alone (6-fold) and the IL-17/TNFα combination (29-fold) compared to the control condition (*p* < 0.05) (Figure 1B). Therefore, the IL-17/TNFα cooperation increased synergistically the secretion of CCL20 and IL-6. By acting on IL-6 and CCL20 secretion, IL-17 and TNFα may contribute to the local Th17 cell induction.

### IL-17 and TNFα Increase ER Stress and Mitochondrial ROS in Myoblasts

**Figure 2:**
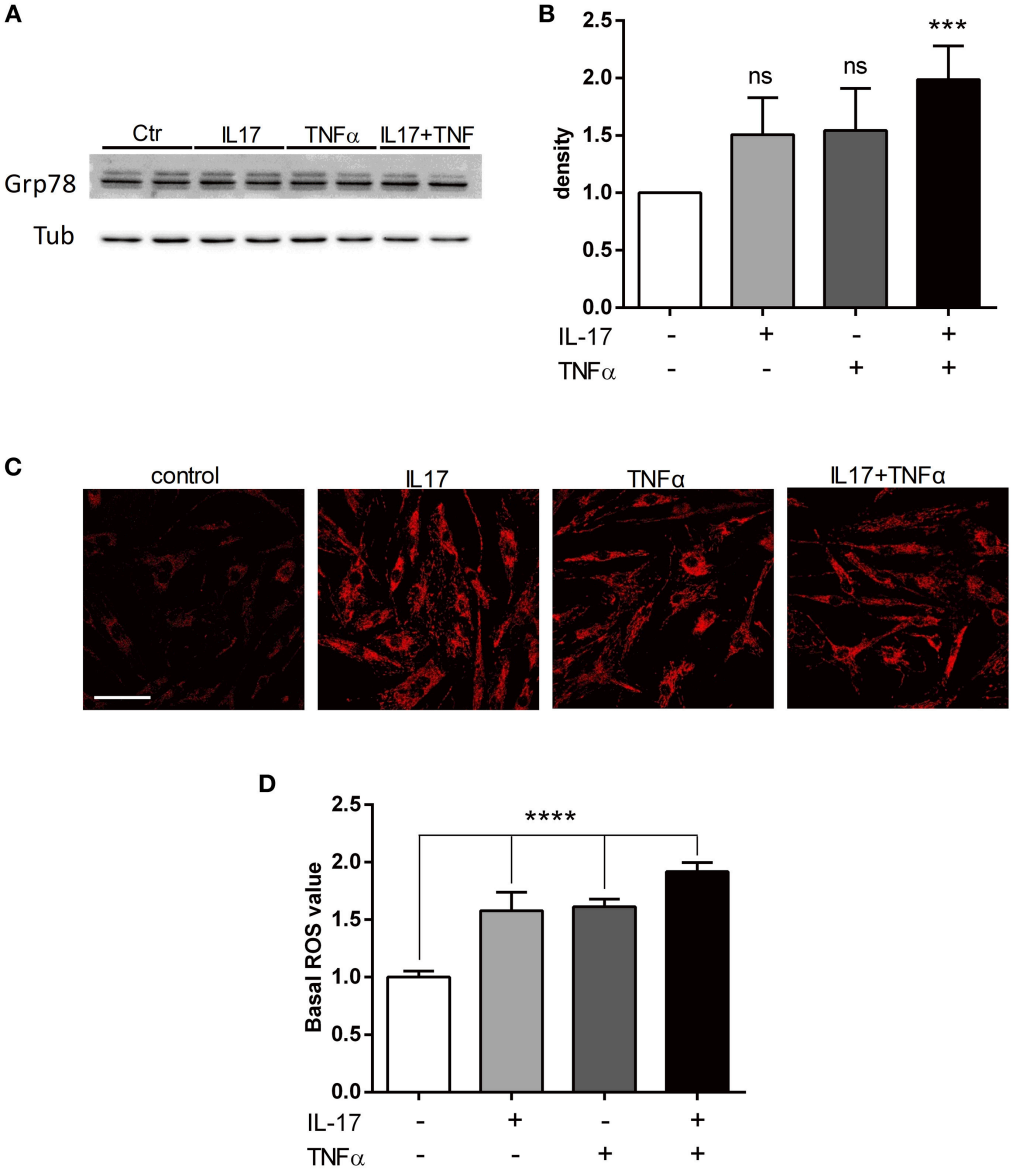
IL-17 and TNFα increase ER stress and mitochondrial ROS in myoblasts. Myoblasts were treated with IL-17 (50 ng/mL) and/or TNFα (1 ng/mL) for 24 h. Expression of BiP/Grp78 protein was measured by western-blot and the band density was normalized with tubulin expression **(A,B)**. Mitochondrial oxidative stress measurements (ROS) of human myoblasts was measured with the fluorescence intensity of CellRox Dye, using 40x objective of a confocal microscope Nikon A1r, scale bar 70 μm **(C,D)**. Data are the mean of 4 to 7 independent experiments ± SEM, ^***^p < 0.001 and ^****^p < 0.0001 vs. control untreated condition.

To study the ER stress triggered by pro-inflammatory cytokine exposure, the expression of BiP/Grp78 protein was quantified. BiP/Grp78 protein controls the activation of the ER stress sensors and initiate the ER stress response known as the unfolded-protein response (19). As observed for the secretion of CCL20 and IL-6, IL-17 and TNFα cooperated to increase the expression of BiP/Grp78 protein compared to IL-17 and TNFα alone (Figures 2A,B). In addition to the unfolded-protein response, oxidative stress and the accumulation of reactive oxygen species (ROS) initiate and contribute to the inflammatory response. Using confocal microscopy, mitochondrial ROS was increased by 1.5-fold in presence of IL-17 or TNFα (*p* < 0.001) alone and by 2-fold in presence of both cytokines (*p* < 0.0001). (Figures 2C,D).

### PBMC-Myoblast Interaction Induces a Strong IL-6 and CCL20 Release

**Figure 3:**
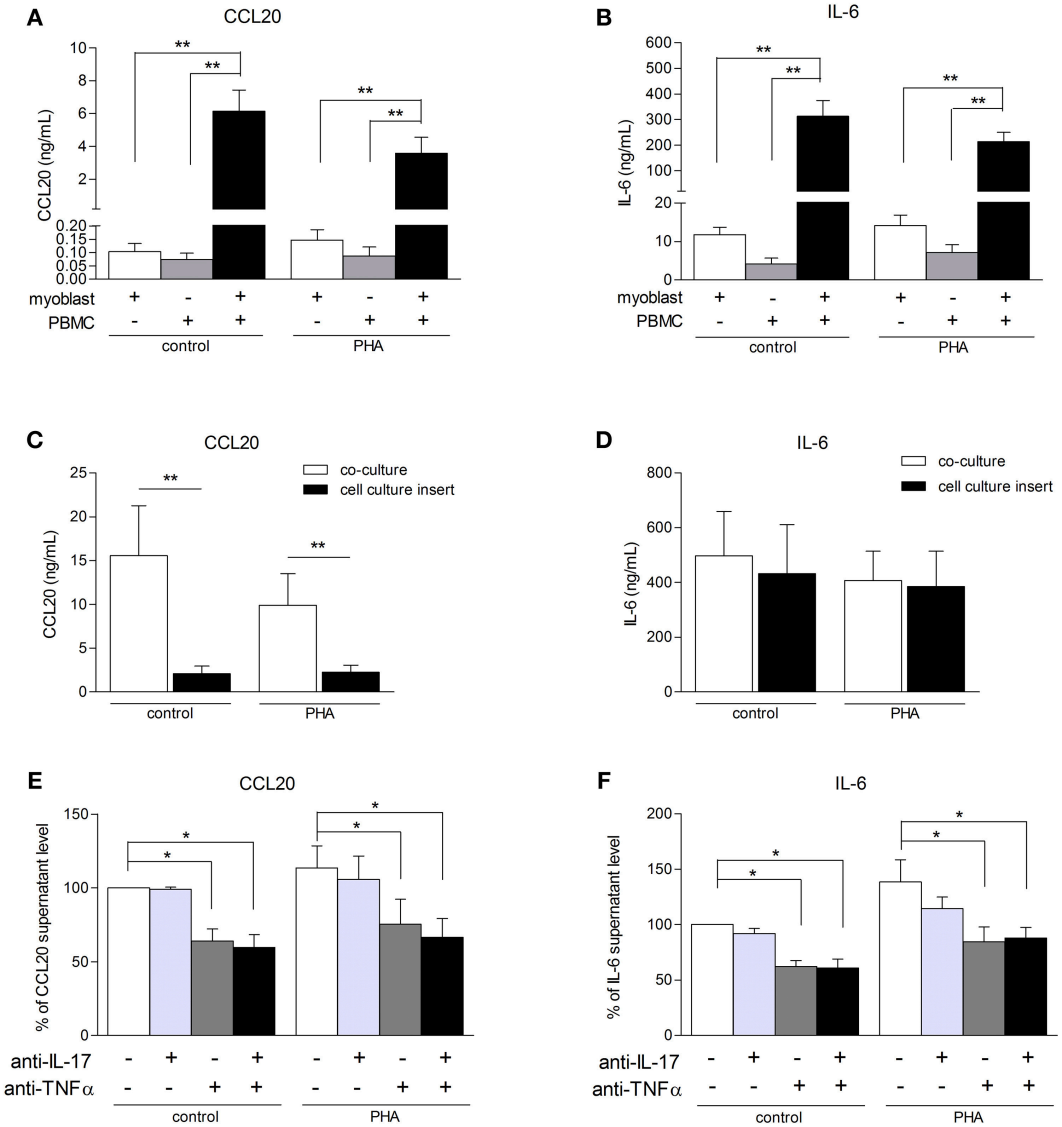
PBMC-myoblast interaction induces a strong production CCL20 and IL-6. PBMC and myoblasts were cultured alone or in co-culture at a ratio of 5 PBMCs for 1 myoblast for 48 h in the presence or not of PHA (5 μg/mL). CCL20 and IL-6 secretion by myoblasts was quantified by ELISA **(A–F)**. The contribution of direct cell-cell contact was investigated with a cell culture permeable insert **(C,D)**. PBMCs were pre-incubated for 24 h in presence or not of PHA and then exposed or not to an anti-IL-17 antibody and/or an anti-TNFα antibody for 3 h before being added to the myoblast cultures. Data are expressed as CCL20 and IL-6 supernatant level percentages compared to the non-activated pre-incubated PBMC—myoblast co-cultures **(E,F)**. Data are the mean of 6–14 independent experiments ± SEM; ^*^p < 0.05, ^**^p < 0.01 vs. control co-culture condition.

To better understand the consequences of immune cell infiltration in muscle tissue of IIM patients (5, 6), a model of co-culture between myoblasts and PBMCs was used. CCL20 and IL-6 were quantified in co-culture supernatants after 48 h. PBMCs from healthy donors were used because it was not possible to perform autologous cultures. In previous studies using mesenchymal cell from skin and synovium, secretion of IL-6, IL-1β, IL-8, and IL-17 were similar between autologous and non-autologous PBMC-mesenchymal cell co-cultures (20–22). The contribution of alloreactivity was minimal compared to the massive effects of cell interactions. In our short-term co-culture models, the PBMC-myoblast interaction induced a strong CCL20 and IL-6 production by comparison to myoblasts alone or PBMCs alone (*P* < 0.01) (Figures 3A,B). PBMC activation with PHA was not required for the increase of CCL20 and IL-6 secretion in co-cultures (Figures 3A–F).

To determine the role of PBMC-myoblast contact in the inflammatory process, transwell cell culture inserts allowing the circulation of soluble factors but not direct cell-cell contact were used. Myoblasts were first added to the bottom of the well and PBMCs to the insert. The use of these inserts reduced strongly CCL20 secretion by 86% in resting co-cultures (*p* < 0.01) and by 77% in PHA-stimulated co-cultures (*p* < 0.01) (Figure 3C). In contrast, the IL-6 release in co-cultures stimulated or not with PHA was not affected (Figure 3D). Therefore, the induction of IL-6 in co-cultures was mainly mediated through soluble mediators between PBMCs and myoblasts.

To determine the contribution of IL-17 and TNFα produced by PBMCs on the CCL20 and IL-6 release in co-cultures, PBMCs activated with PHA for 24 h were exposed to specific inhibitors of IL-17 and/or TNFα and then added to myoblast cultures. This PBMC pre-incubation step was used to better mimic the *in vivo* conditions in chronic inflammatory state. As shown in Figures 3E,F, neutralization of IL-17 did not reduce the CCL20 and IL-6 secretion in our co-culture system. By contrast, the anti-TNFα antibody inhibited the CCL20 and IL-6 production both in unstimulated condition (36 and 42% of inhibition, respectively, *p* < 0.05) and PHA condition (34 and 35% of inhibition, respectively, *p* < 0.05). Moreover, the use of both anti-IL-17 and anti-TNFα decreased also significantly the CCL20 and IL-6 secretion without additive or synergistic inhibitory effects (Figures 3E,F). Therefore, TNFα contributed to the induction of CCL20 and IL-6 release in PHA-activated co-cultures.

### IL-17 and TNFα Increase Store Operated Calcium Entry (SOCE)

Inflammation increases intracellular Ca^2+^ concentrations in several inflammatory muscle disorders. The routes of calcium entry include calcium leak channels, stretch-activated channels, receptor-operated channels, and store-operated calcium channels. Ca^2+^ influx is sufficient to induce muscular dystrophy through a TRPC-dependent mechanism (23). The pro-inflammatory cytokine TNFα has been shown to enhance SOCE in human airway smooth muscle cells (24). The effect of IL-17 and TNFα on the calcium homeostasis has not been investigated in the context of IIM pathogenesis.

**Figure 4:**
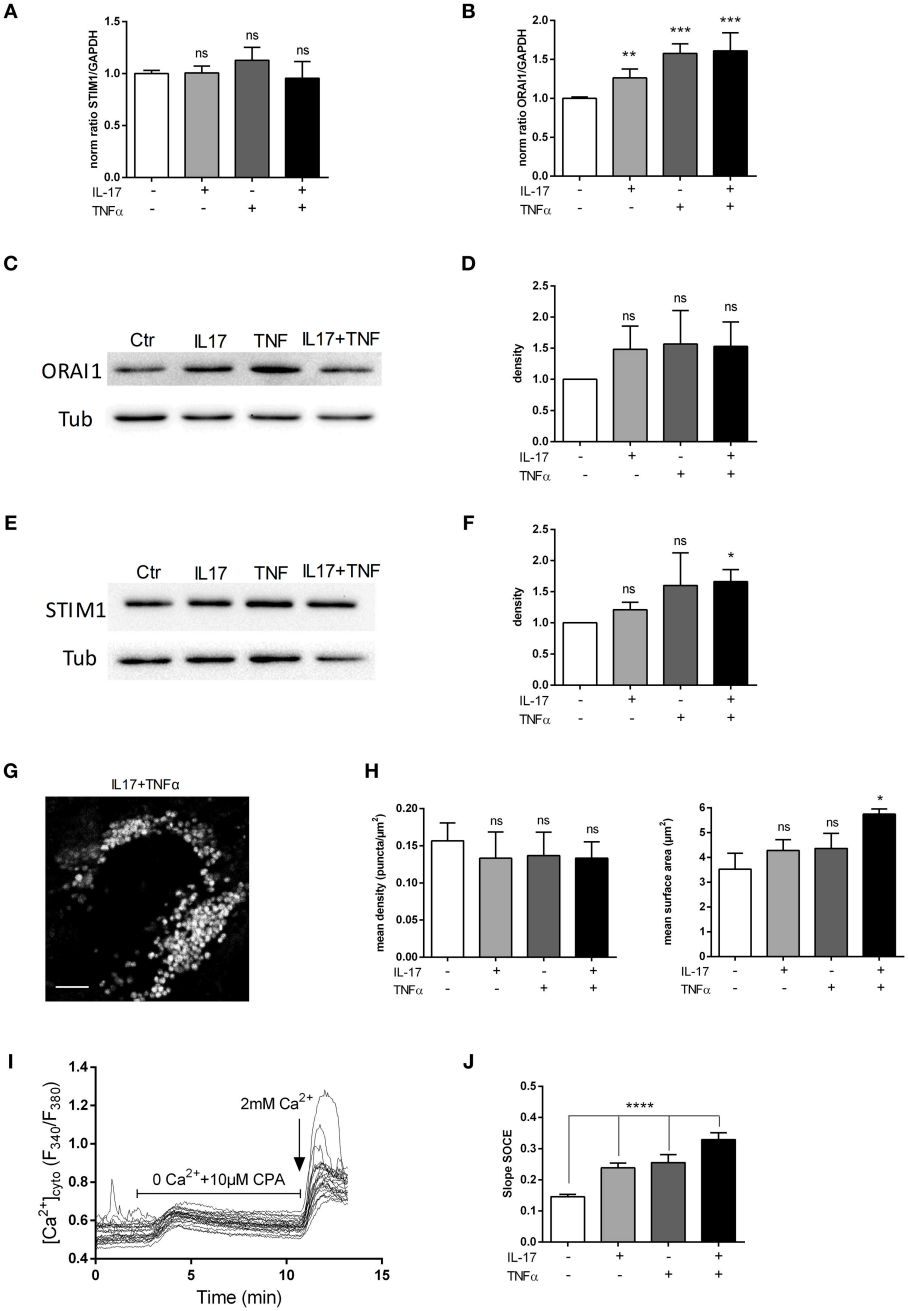
IL-17 and TNFα increase store-operated calcium entry. Myoblasts were treated with IL-17 (50 ng/mL) and/or TNFα (1 ng/mL). mRNA levels of STIM1 and ORAI1 at 12 h was expressed as fold changes compared to control **(A,B)**. ORAI1 and STIM1 protein was measured by western-blot and the band density was normalized with the tubulin expression. **(C–F)** Representative image of STIM1 puncta in human myoblast treated with IL-17 (50 ng/mL) and TNFα (1 ng/mL) for 24 h. Image Correlation Spectroscopy (ICS) analysis of STIM1 puncta (left inset) mean density of puncta (μm2). (right inset) mean surface of puncta (puncta/μm2). Data are the mean of 3 independent experiments with cells from 3 different donors ± SEM; ^*^p < 0.05 vs. control untreated condition, scale bar 3 μm **(G,H)**. SOCE was measured by using Fura2-AM dye in human myoblasts. Cells were imaged on an epifluorescence microscope using a 40x objective. Trace is a representative measurement of SOCE in IL17/TNFα treated myoblasts. **(I)** Slope analysis of the SOCE **(J)** Data are the mean of 4–5 independent experiments ± SEM; ^*^p < 0.05 and ^****^p < 0.00001, vs. control untreated condition. ^*^p < 0.05; ^**^p < 0.01; ^***^p < 0.001.

Orai1 and STIM1 mRNA levels were first measured in human myoblasts after 6 and 12 h of IL-17 and/or TNFα exposure. No significant effect in mRNA levels of ORAI1 and STIM1 was detected at 6 (data not shown) and 12 h (Figures 4A,B). Orai1 and STIM1 protein levels were next investigated by western blot. IL-17/TNFα increased significantly STIM1 expression compared to control (Figures 4E,F), whereas no significant increase in ORAI1 expression was observed (Figures 4C,D). STIM1 puncta formation analysis revealed no significant difference in the puncta density, but an increase of STIM1-puncta surface in IL-17/TNFα conditions (Figures 4G,H).

To confirm the effect of cytokines on SOCE, a fluorescence-based measurement of SOCE in human myoblasts was performed after cytokine treatment. IL-17 and TNFα single treatment modified the slope of SOCE by 1.4-fold and the IL-17/TNFα combination by 2-fold compared to control condition (*p* < 0.01) (Figures 4I,J).

### Inhibition of SOCE Reduces IL-6 Production Induced by the IL-17/TNFα Combination

**Figure 5:**
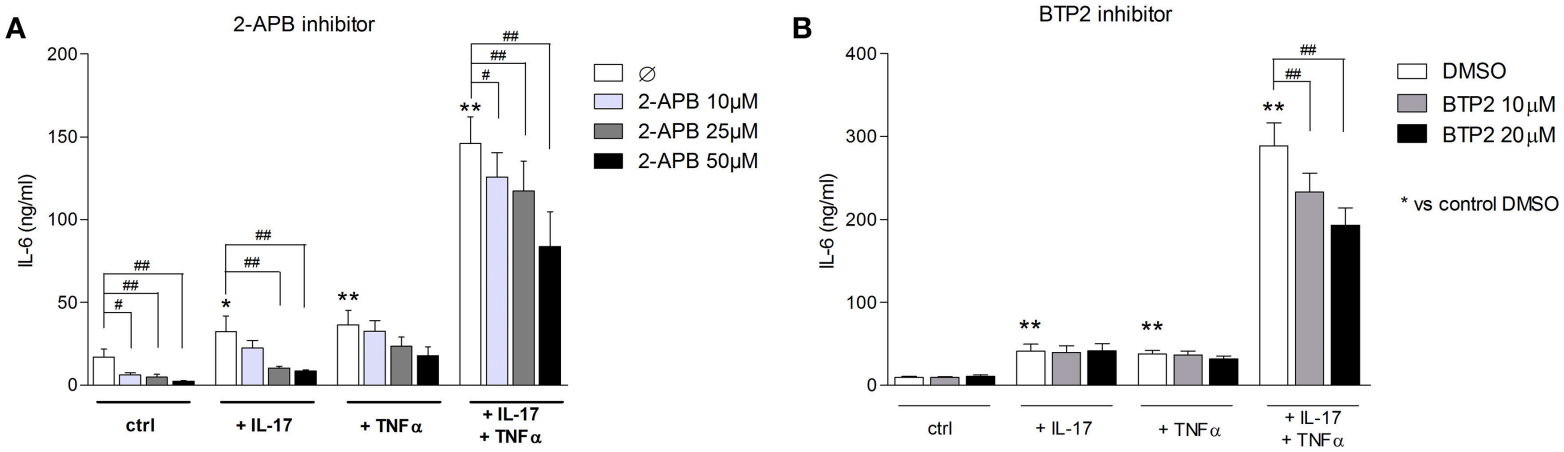
Inhibition of SOCE reduces IL-6 production induced by the IL-17/TNFα combination. Myoblasts were stimulated with IL-17 and/or TNFα in presence or not of the SOCE inhibitor 2-APB (10, 25, and 50 μM) or BTP2 (10 and 20 μM) for 48 h. CCL20 and IL-6 secretion by myoblasts was quantified by ELISA **(A,B)**. Data are the mean of 7 independent experiments ± SEM; ^**^p < 0.01 vs. control condition [medium alone **(A)** and medium+DMSO **(B)**] and #*p* < 0.05, ##*p* < 0.01 vs. other inflammatory conditions.

To assess the SOCE contribution to the myoblast inflammatory response, myoblasts were stimulated with IL-17 and/or TNFα in presence or not of 2-APB or BTP2 SOCE inhibitors. IL-6 release by myoblasts was quantified at 48 h. The 2-ABP and BTP2 inhibitors inhibited the induction of IL-6 production by IL-17 and/or TNFα in a dose-dependent manner. The induction of IL-6 secretion by the IL-17 and TNFα combination was reduced by 42% (*p* < 0.05) with 2-ABP at 50 μM, and by 19% (*p* < 0.05) and 33% (*p* < 0.01) with BTP-2 at 10 and 20 μM, respectively (Figures 5A,B). Therefore, SOCE interacts with immune mechanisms to further increase the myoblast inflammatory response.

## Discussion

Both immune and non-immune mechanisms contribute to IIM pathogenesis. The interplay between these two mechanisms was studied in human myoblasts stimulated with the pro-inflammatory cytokines IL-17 and TNFα. Immature muscle precursors are immunologically active cells, playing an important role in disease progression and probably in muscle regeneration defects observed in IIM patients (16, 25, 26). The results indicate that in addition of the inflammatory response induced by IL-17 and TNFα; IL-17 and TNFα promoted also ER and mitochondrial stress and calcium dysregulation in myoblasts, leading to muscle cell dysfunction.

The immune cell infiltrate contributes to the pathogenesis of IIM through cell-cell interactions and the release of cytokines. Myoblasts may contribute to mononuclear cell attraction by secreting chemokines in response to local inflammation (25). CCL20 expression was found in dermatomyositis and polymyositis muscle samples and was associated with dendritic and Th17 cell homing (11). In this study, IL-17 and TNFα had a cooperative synergistic effect on CCL20 secretion by isolated myoblasts. These results are in line with our previous reports indicating that IL-17 increases TLR-3 agonist and IL-1β-induced CCL20 production by myoblasts (10, 16). Therefore, IL-17 can exacerbate the weak effects of low concentrations of TNFα and IL-1β on CCL20 release. Because CCL20 induces Th17 and dendritic cell recruitment, this local increase of CCL20 could contribute to the induction and perpetuation of the IIM local inflammation.

IL-6 is a pleiotropic inflammatory cytokine produced by myoblasts in response to inflammation. The IL-17/IL-1β, TNFα/IL-1β, and TNFα/IFNγ combinations have been previously shown to have additive/synergistic effects on the induction of IL-6 secretion by myoblasts (10, 15, 25, 27). Here, IL-17 and TNFα interactions increased in synergy the production of IL-6.

To mimic the *in vivo* environment characterized by immune cell infiltration in IIM, a PBMC and myoblast co-culture system was used. The interactions between PBMCs and myoblasts induced a strong CCL20 and IL-6 release and PBMC activation with PHA was not required for this induction. In synoviocyte-PBMC or skin fibroblast-PBMC co-cultures, cell interactions were also sufficient to induce the IL-6 or IL-8 secretion (21, 22). The increase of CCL20 production in co-cultures was mediated through direct PBMC-myoblast contacts since the use of cell culture insert reduced strongly its production in co-culture. Another study showed that the T cell-myoblast direct interactions can lead to T cell proliferation (28). By contrast, the IL-6 secretion was mainly induced through soluble factors in myoblast-PBMC co-culture whereas the direct cell contact was significantly involved in IL-6 generation in synoviocyte-PBMC or skin fibroblast-PBMC co-cultures (21, 22). Neutralization of IL-17 and/or TNFα in the co-culture system with pre-incubated PBMCs and myoblasts reduced IL-6 release, confirming the contribution of the soluble factors. Therefore, the soluble inflammatory cytokines IL-17 and TNFα could have an important role in initiating and maintaining inflammation *in vivo* in IIM through the production of IL-6, which then contributes to Th17 cell differentiation.

The ER stress pathways are activated in tissues from patients with IIM (29, 30) and interplay with mitochondrial dysfunction and ROS generation (31). In myoblasts, we identified that IL-17 and/or TNFα mediated ER stress and mitochondrial ROS, suggesting that these cytokines may participate *in vivo* to these non-immune mechanisms. Moreover, ER is the main intracellular Ca^2+^ storage, and ER stress induces Ca^2+^ ER release. Changes in Ca^2+^ homeostasis can affect muscle contractibility (32). Ca^2+^ dysregulation was reported in sporadic inclusion body myositis (33). SOCE is a key component of the intracellular calcium concentration and plays an important role in muscle function and development (2, 4, 34). SOCE is modified during inflammation (24, 35). In this study, IL-17 and/or TNFα exposure increased SOCE in myoblasts with a higher effect when IL-17 and TNFα were combined. In human airway smooth muscle cells, TNFα enhanced Orai1, STIM1, and SOCE (35). In myoblasts, TNFα alone did not increase significantly Orai1 and STIM1 expression but the concentration was 20-fold lower. However, the IL-17/TNFα combination increased significantly STIM1 protein level as well as STIM1-puncta surface in myoblasts but had no significant effect on STIM1 and Orai1 mRNA expression or Orai1 protein level. TNFα and IL-13 have been shown to increase STIM1 aggregation in human airway smooth muscle cells, contributing to SOCE induction (24). Therefore, IL-17 and/or TNFα may increase SOCE by inducing Ca^2+^ release from ER and enhancing STIM1 aggregation.

**Figure 6:**
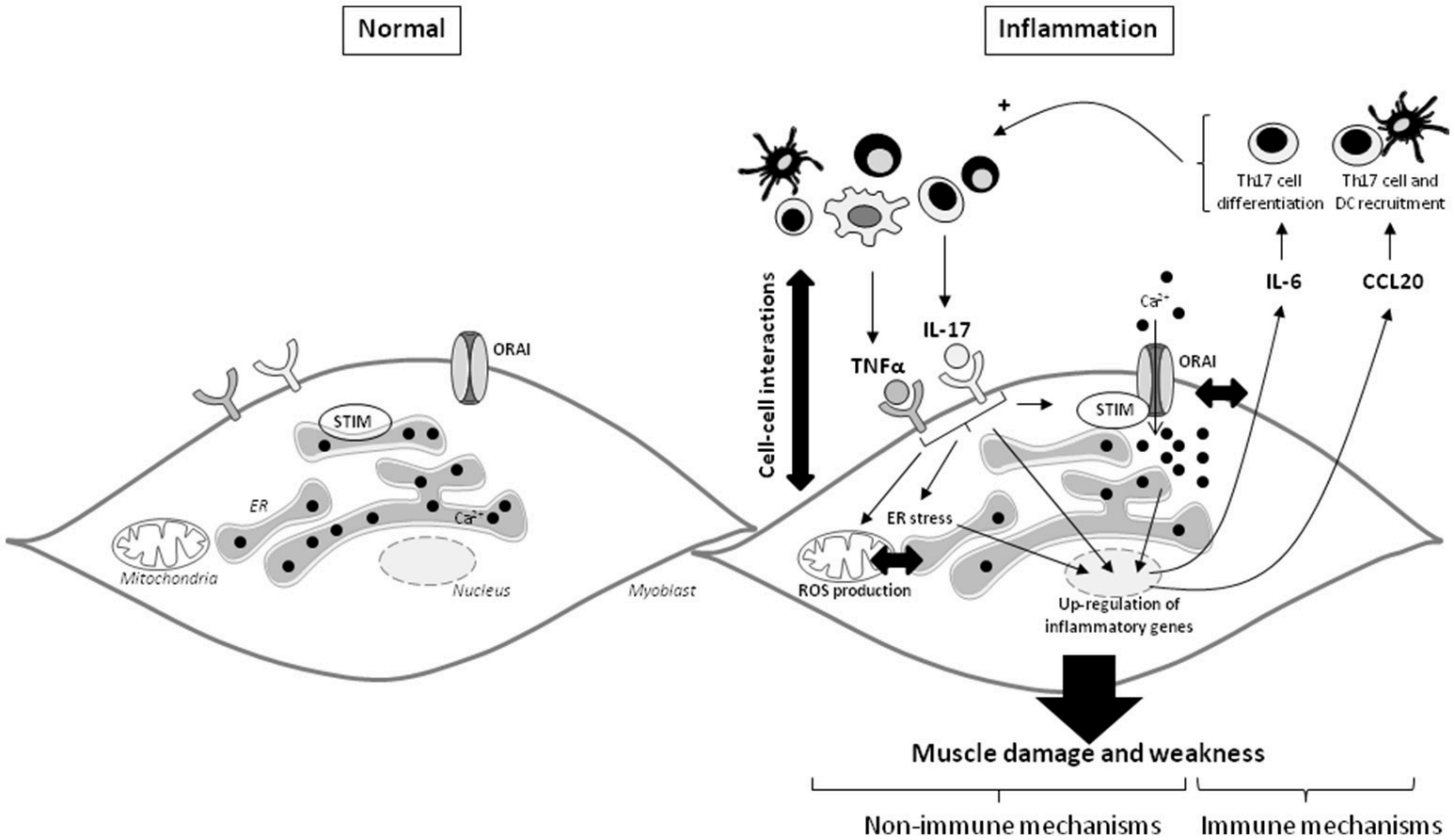
IL-17 and TNFα mediate muscle damage and weakness through immune and non-immune pathways in myoblasts. The immune cell infiltration in IIM constitutes a local source of cytokines and promotes the cell-cell interactions. IL-17 mainly produced by Th17 cells, and TNFα act in synergy on myoblasts to increase IL-6 and CCL20 secretion. Because IL-6 is involved in the Th17 cell differentiation and CCL20 in dendritic and Th17 cell recruitment, IL-6 and CCL20 mediate a positive feedback loop promoting local IL-17 production. IL-17 and TNFα induce also non-immune pathways with ROS production, ER stress and SOCE activation. The IL-17/TNFα effect of mitochondrial dysfunction, ER stress, and SOCE activation are probably closely linked. SOCE and calcium dysregulation contribute to the IL-6 release induced by IL-17/TNFα. CCL20, chemokine (C-C motif) ligand 20; DC, dendritic cells; ER, endoplasmic reticulum; IL, interleukin; ROS, reactive oxygen species; STIM, stromal interacting molecule; TNFα, tumor necrosis factor-α.

To determine the role of SOCE in the inflammatory response induced by IL-17/TNFα, SOCE inhibitors were used. SOCE inhibition reduced the secretion of IL-6 following IL-17/TNFα exposure. In human bronchial epithelial cells, SOCE inhibition with BTP2 inhibited IL-6 and IL-8 production after allergen stimulation (36). Therefore, in addition to muscle cell dysfunction, SOCE may have a central role in the induction and maintenance of the inflammatory state. Its neutralization could be a promising therapeutic strategy in IIM. These results in myoblasts are summarized in Figure 6.

## Author Contributions

AB, YG, and FL: experiments, assay development, and writing; PM and MO: concept, supervision, and writing.

### Conflict of Interest Statement

The authors declare that the research was conducted in the absence of any commercial or financial relationships that could be construed as a potential conflict of interest.
